# An Unusual Case of Bicuspid Aortic Valve With Prolapse Masquerading as Cusp Perforation

**DOI:** 10.7759/cureus.60562

**Published:** 2024-05-18

**Authors:** Vikas Kilaru, Sharvil Patel, Hely Patel, Nima Ghasemzadeh

**Affiliations:** 1 Internal Medicine, Northeast Georgia Medical Center Gainesville, Gainesville, USA; 2 Pediatrics, Emory University, Atlanta, USA; 3 Interventional Cardiology, Georgia Heart Institute, Gainesville, USA

**Keywords:** aortic valve prolapse, acute onset severe hypoxic respiratory failure, transthoracic echocardiogram(tte), cardiogenic shock, surgical aortic valve replacement (savr), bicuspid aortic valve, aortic valve insufficiency

## Abstract

We present a unique clinical scenario of a 58-year-old male with a past medical history of hypertension who initially presented with chest pain and was ruled in for non-ST elevation myocardial infarction (NSTEMI) but rapidly developed respiratory failure secondary to aortic insufficiency complicated by cardiogenic shock (CS), attributed to aortic valve prolapse. Intriguingly, the patient had a normal ECG on presentation, underscoring the dynamic nature of valvular pathology. The development of CS highlights the importance of early recognition, prompt diagnosis, and interdisciplinary management in such complex cases.

## Introduction

Aortic insufficiency (AI) is characterized by the retrograde flow of blood from the aorta into the left ventricle during diastole [1]. In undifferentiated patients, the presence of significant valvular disease can present a unique challenge, especially when initial diagnostic imaging and evaluation are unrevealing. In addition, the severity of AI can be magnified in patients with aortic valve prolapse (AVP) who have concomitant bicuspid aortic valve (BAV) pathology [2]. Previous literature has also shown that the degree of AVP was significantly greater for patients with BAV [3]. While AVP is an infrequent etiology of AI, its presence can manifest in a variety of ways, including significant hemodynamic compromise and cardiogenic shock (CS). In this report, we describe the case of a patient with hypertension who initially presented with non-ST elevation myocardial infarction (NSTEMI) but rapidly progressed to respiratory failure and CS due to AI secondary to AVP.

Despite the absence of prior cardiac history, the swift decline experienced by this patient underscores the necessity for consideration of less frequent pathology. The case emphasizes the dynamic nature of valvular pathology and the potential for severe complications, necessitating a multidisciplinary approach for timely diagnosis and management.

## Case presentation

**Figure 1:**
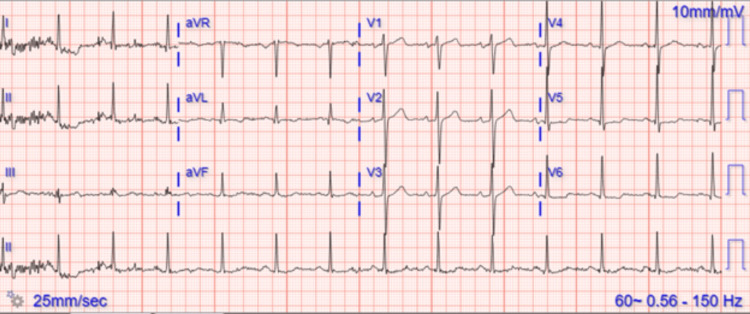
ECG with ST depressions in leads 1 and aVL

A 58-year-old male with a history of hypertension presented to the emergency department with chest discomfort and shortness of breath. He had no prior cardiac history. His vitals were notable for a BP of 143/48 mmHg, a heart rate of 87 bpm, and a temperature of 98.7 °F. Arterial blood gas showed pO2 of 58.6 mmHg, pCO2 of 37.4 mmHg, pH 7.37, and lactate of 1.40 mg/dL. On arrival, he was diagnosed with NSTEMI. His ECG was concerning for lateral ischemia (Figure 1), and he subsequently underwent cardiac catheterization, which showed no significant epicardial coronary artery disease.

He was additionally found to have community-acquired pneumonia and was started on empiric antibiotics with ampicillin-sulbactam 3 g IV q6h and azithromycin 500 mg IV, followed by 250 mg IV daily for four days. A transthoracic echocardiogram (TTE) was performed at arrival and was largely unremarkable, revealing mild AI and a normal left ventricular ejection fraction, lowering suspicion of any cardiac issues. Laboratory values on arrival and during respiratory distress 18 hours later are reported in Table 1.

**Table 1:** Significant laboratory values at presentation vs. after decompensation LVEF, left ventricular ejection fraction; PLT, platelet

Laboratory tests	Initial values	Values after decompensation	Reference range
Sodium (mmol/L)	141	136	135-148
Potassium (mmol/L)	4.0	4.1	3.5-5.2
Creatinine (mg/dL)	1.06	1.39	0.80-1.30
WBC (K/uL)	9.9	20.9	4.8-10.8
PLT (K/uL)	272	272	130-400
HS troponin I (ng/L)	97	1,442	<45
Hemoglobin (g/dL)	16.5	17.0	14.0-18.0
AI grade	Trace regurgitation	Severe regurgitation	-
LVEF	60-65%	55-60%	50-70%

Over the next 18 hours, the patient’s respiratory status deteriorated rapidly, with severe dyspnea and desaturations. On physical examination, he was in respiratory distress, with wheezing on lung auscultation. He was subsequently intubated and paralyzed, and central access was established for intravenous pressors due to hypotension. Both sets of blood cultures obtained prior to the initiation of antibiotics were negative at 120 hours. Due to ongoing hypoxemic respiratory failure and frequent desaturations, the patient was transferred to an extracorporeal membrane oxygenation (ECMO)-capable center for further management. Serial chest X-rays showed diffuse bilateral infiltrates and suspicion of fluid overload, resulting in initial diuresis, which was escalated. Antibiotics at this time were expanded to vancomycin, renally dosed, and cefepime 2 g IV q8h.

**Figure 2:**
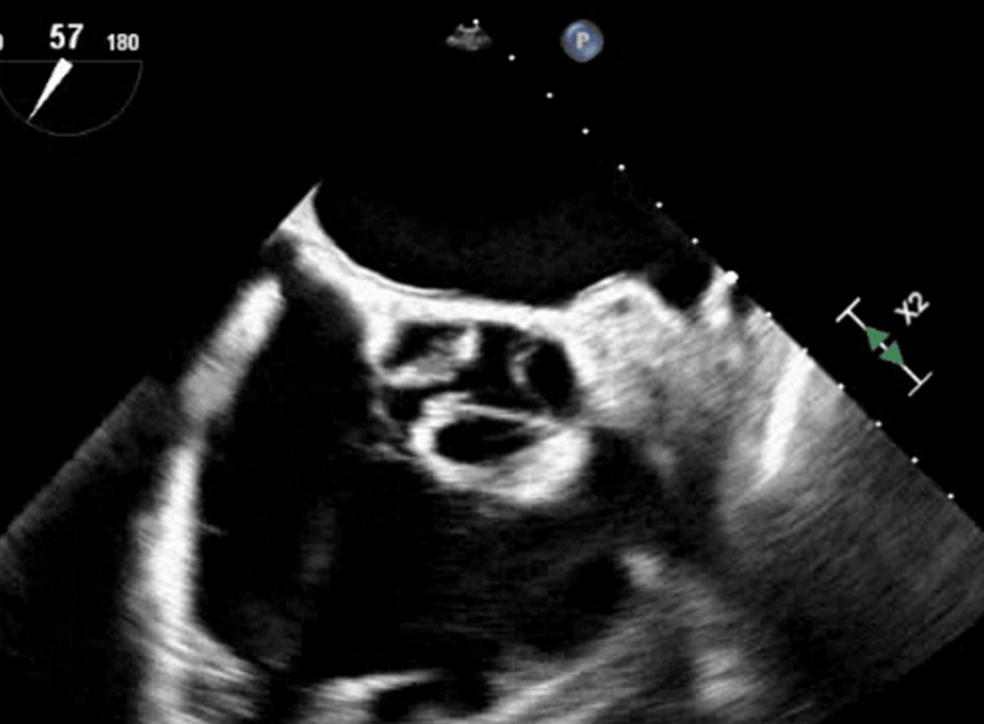
BAV seen on TEE with possible perforated left cusp and vegetation BAV, bicuspid aortic valve; TEE, transesophageal echocardiogram

**Figure 3:**
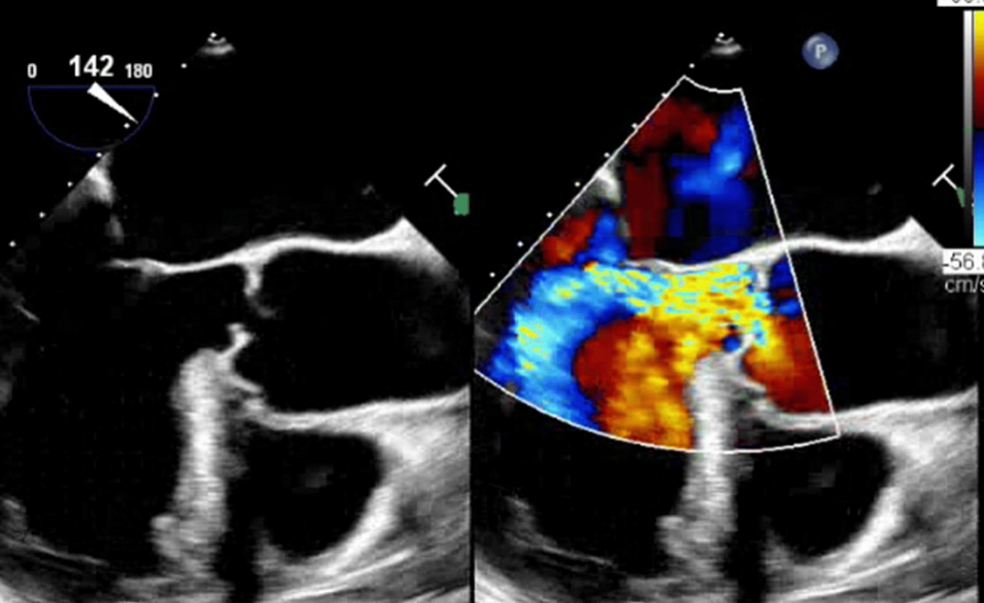
Aortic valve seen on a TEE, demonstrating severe AI and AVP AI, aortic insufficiency; AVP, aortic valve prolapse; TEE, transesophageal echocardiogram

TTE was repeated five days after the first due to a lack of improvement as well as suspicion of a new Austin Flint murmur, demonstrating severe aortic regurgitation along the anterior mitral leaflet. A transesophageal echocardiogram (TEE) confirmed severe AI, as well as possible perforated left cusps and likely small vegetation (Figures 2, 3).

Cardiothoracic surgery was consulted and eventually elected for intervention due to suspected endocarditis. During surgery, AVP was identified as the underlying etiology, and fusion of the left and right coronary cusps was identified. The leaflet tissue was sent for microbiologic evaluation and was negative for any infection. Aortic valve replacement was performed with a follow-up echo, revealing a well-seated valve with no significant paravalvular leaks. The patient had a prolonged postoperative course due to pneumonia, renal failure, and CS but demonstrated good recovery and was discharged with plans for subacute and cardiac rehabilitation.

## Discussion

The aortic valve represents a complex anatomical and functional structure susceptible to a wide variety of pathologies, including congenital anomalies like the BAV. BAV is the most common congenital heart defect in which the aortic valve contains two leaflets instead of three. It is generally estimated to exist in 0.5 to 1.4% of the population [1]. In many cases, it remains undetected until the abnormal flow patterns created by the bicuspid valve lead to progressive calcification, resulting in severe valvular dysfunction in the form of aortic stenosis (AS) or AI. It is also commonly associated with infective endocarditis and aortic dissection [4]. In rare cases, it can ultimately lead to AVP, which is characterized by the downward displacement of one or more aortic valve cusps into the left ventricular outflow tract during diastole. The incidence of AVP is about 1%, and approximately 30% of the patients with AVP had a BAV [5]. AVP can be best diagnosed on a TEE, but it also requires analysis by surgical inspection. The etiology of AVP varies and can be attributed to inflammatory degenerative changes, trauma, infection, or idiopathic causes [6]. Although AVP does not necessarily imply the presence of AI, it may precipitate its development with concurrent BAV [2].

While AS is a common downstream effect of BAV, the possibility of acute AI must be considered. The development of AI in this setting is typically multifactorial. Chronic valvular degeneration, characterized by fibrosis, calcification, and thickening of the leaflets, compromises valvular integrity [7]. These abnormalities of the valve can predispose it to malcoaptation, resulting in altered valve closure and retrograde flow into the left ventricle. While chronic AI allows the ventricle time to adapt to increased volume, a sudden increase in regurgitant volume results in significantly increased left ventricular diastolic pressure and increased afterload, resulting in a sharp fall in left ventricle output [8,9]. In patients with left ventricular hypertrophy, such as those with chronic hypertension, this process can be even more conspicuous [10]. The combination of these deleterious effects leads to distal tissue hypoperfusion, ineffective arterial blood volume, increased atrial and pulmonary vein pressures, hypotension, progressive CS, and respiratory failure. Alternatively, catheter-induced trauma to the aortic valve leaflets or annulus could result in valvular insufficiency, resulting in worsening heart failure symptoms and sudden cardiac decompensation [11-13]. While iatrogenic aortic regurgitation may arise post-cardiac catheterization due to procedural trauma, there is a notable absence of cases concerning the development of AVP in this context.

CS is a physiologic state of cardiac dysfunction characterized by tissue hypoperfusion and diminished efficient cardiac output, with significant associated mortality. CS secondary to BAV and AVP represents a rare but clinically significant manifestation of structural heart disease requiring prompt recognition and intervention (CS with BAV and AVP prevalence). Medical management in the case of CS due to, or in the presence of, aortic valve insufficiency is limited. There is also a paucity of guidance in the literature on optimal therapy in this clinical conundrum. Intra-aortic balloon pump would be contraindicated due to inflation of the balloon in diastole increasing regurgitation into the left ventricle, and Impella is relatively contraindicated due to concern for increased aortic pressure. While the use of ECMO has quadrupled in the past 10 years, venoarterial ECMO is limited in utility due to the implication of increased ventricular afterload [14]. CS can be further complicated by the presence of sepsis, and acute coronary syndrome (ACS)-associated CS with concomitant sepsis has been shown to have twice the risk of mortality [15]. Younger age and decreased systemic vascular resistance were significant indicators of prolonged treatment and persistence of CS. Replacement of the aortic valve in the setting of CS secondary to AI continues to be the focus of treatment [16]. While evidence is growing that a transcatheter aortic valve is a safe and viable option, the standard of care remains surgical aortic valve replacement [17]. In this case, our patient significantly benefited from early surgical intervention, and recovery was achieved with the aid of aggressive inotropic and vasopressor therapy.

Differential diagnosis

Our differential diagnosis for this patient included endocarditis with aortic cusp perforation, acute respiratory distress syndrome (ARDS), CS, ACS, and pulmonary embolism. Given the degree of respiratory failure requiring intubation, pulmonary etiology remained of high suspicion. A computed tomography pulmonary angiogram was performed without evidence of embolism but did reveal significant bilateral infiltrates. Leukocytosis and fever were present, increasing the likelihood of an infectious process, although cultures remained negative. Lack of improvement with traditional ARDS management and antibiotics led to repeat TTE and subsequent TEE, revealing significant AI along with rapid decompensation leading to CS.

## Conclusions

This case underscores the importance of considering acute AI, especially when related to AVP, as a potential cause of CS and respiratory failure, even if initial diagnostics are not suggestive of such. As presented, acute AI can evolve quickly, and timely diagnosis and potentially surgical intervention are paramount to prevent adverse outcomes in such complex cases. An integrated cross-disciplinary approach is needed for the proper care of these patients given the fluctuating characteristics of valvular conditions, as exemplified in this case.
